# Physiological Responses at Rest and Exercise to High Altitude in Lowland Children and Adolescents

**DOI:** 10.3390/life11101009

**Published:** 2021-09-24

**Authors:** Morin Lang, Guillem Vizcaíno-Muñoz, Paulina Jopia, Juan Silva-Urra, Ginés Viscor

**Affiliations:** 1 Department of Rehabilitation Sciences and Human Movement, Faculty of Health Sciences, University of Antofagasta, Antofagasta 1240000, Chile; 2 Physiology Section, Department of Cell Biology, Physiology and Immunology, Faculty of Biology, Universitat de Barcelona, E-08028 Barcelona, Spain; guillebike96@hotmail.com (G.V.-M.); gviscor@ub.edu (G.V.); 3 Occupational Health Department, Institute of Occupational Safety, Copiapó 1530000, Chile; paulina.jopia@ist.cl; 4 Biomedical Department, Faculty of Health Sciences, University of Antofagasta, Antofagasta 1240000, Chile; juan.silva.urra@uantof.cl

**Keywords:** high altitude, acute hypoxia, children, altitude acclimatization, acute mountain sickness (AMS)

## Abstract

During the last decades, the number of lowland children exposed to high altitude (HA) has increased drastically. Several factors may influence the development of illness after acute HA exposure on children and adolescent populations, such as altitude reached, ascent velocity, time spent at altitude and, especially, their age. The main goal of this study was to evaluate the resting cardiorespiratory physiological and submaximal exercise responses under natural HA conditions by means of the six-minute walking test (six MWT). Secondly, we aimed to identify the signs and symptoms associated with acute mountain sickness (AMS) onset after acute HA exposure in children and adolescents. Forty-two children and adolescents, 18 boys and 24 girls aged from 11 to 15 years old, participated in this study, which was performed at sea level (SL) and during the first 42 h at HA (3330 m). The Lake Louise score (LLS) was recorded in order to evaluate the evolution of AMS symptoms. Submaximal exercise tests (six MWT) were performed at SL and HA. Physiological parameters such as heart rate, systolic and diastolic blood pressure, respiratory rate and arterialized oxygen saturation were measured at rest and after ending exercise testing at the two altitudes. After acute HA exposure, the participants showed lower arterial oxygen saturation levels at rest and after the submaximal test compared to SL (*p* < 0.001). Resting heart rate, respiratory rate and diastolic blood pressure presented higher values at HA (*p* < 0.01). Moreover, heart rate, diastolic blood pressure and dyspnea values increased before, during and after exercise at HA (*p* < 0.01). Moreover, submaximal exercise performance decreased at HA (*p* < 0.001). The AMS incidence at HA ranged from 9.5% to 19%, with mild to moderate symptoms. In conclusion, acute HA exposure in children and adolescent individuals produces an increase in basal cardiorespiratory parameters and a decrement in arterial oxygen saturation. Moreover, cardiorespiratory parameters increase during submaximal exercise at HA. Mild to moderate symptoms of AMS at 3330 m and adequate cardiovascular responses to submaximal exercise do not contraindicate the ascension of children and adolescents to that altitude, at least for a limited period of time.

## 1. Introduction

During the last decades the number of lowland children exposed to high altitude (HA) has increased drastically [1]. The main reasons not only include the increase in transient trips to mountain resorts or academic activities but also include children moving with their families to HA areas due to parent’s job changes or even increased migration across borders of refugee families from neighboring countries in high-altitude crossings. During the rapid ascension from lowlands altitudes to HA, subjects experienced a drastic drop in environmental partial pressure of oxygen and a reduction in barometric pressure with the increase in altitude [2].

The decrease in barometric environmental pressure and the consequent decrease in partial pressure of oxygen caused a state of reduced oxygen availability, known as hypobaric hypoxia [3]. Hypobaric hypoxia is one of the main factors why children lose their usually active behavior during acute HA exposure after rapid ascent to altitudes above 2500 m [4].

Moreover, there is enough evidence to affirm that children are even more susceptible than adults to a wide range of altitude associated illness, such as acute mountains sickness (AMS), high-altitude cerebral edema or high-altitude pulmonary edema [5]. Among these symptoms, the development of AMS stands out; this appears on children after a rapid ascent at altitudes over 2500 m, taking into consideration the onset of headache, fatigue, dizziness, sleeping difficulties, anorexia, nausea, vomiting and changes in mental status [5,6]. AMS is frequently associated with the development of dyspnea, tachypnea, decreased tolerance to activity, cyanosis, cough and fever [5]. These symptoms usually appear between 4 and 8 h after exposure to HA, however, they can also present beyond 96 h after arrival [7]. 

Some studies report that the incidence of AMS in child populations appears to be similar to that observed on adults [8,9,10,11]. However, these studies were performed on children that permanently live at high altitude locations, from 3006 m to 4380 m, where average children did not have the opportunity to arrive. Therefore, these results cannot be useful for assessing children traveling from lowland cities. In addition, a recent meta-analysis suggests that there is no association between age and risk of AMS [12]. Few studies suggest that child populations could be more susceptible to AMS than the adult population [13]. Other studies indicates that the prevalence of AMS before puberty and in adolescence might be slightly less than in adults [14].

On the other hand, experimental studies in prepubertal male rats showed the smallest tolerance to hypoxia correlated with inflammatory diseases [15]. Indeed, a recent review has shown that the hypoxia-inducible factor (HIF)-1 expression levels’ variability, determined by age, gender, ethnicity and others, could predispose the individual in terms of tolerance and adaptation to hypoxia and development of certain infectious inflammatory and tumor processes [16]. 

Gender does not have a clear effect on AMS risk. Some studies have found no differences between females and males [17,18]. While a study has shown that females present a higher prevalence of AMS [8], other studies indicate that males present higher AMS than females [19].

Acute exposure to high altitude causes changes in cardiovascular activity driven by strong adrenergic responses dependent on central and peripheral arterial chemoreceptors. The primary cardiovascular response is characterized by an increase in cardiac output by increasing the heart rate (tachycardia) and a transitory rise in blood pressure in order to balance the decreased stroke volume [2]. Consequently, acute environmental hypoxia is counteracted mainly by increasing cardiac output and raising the ventilation rate [20].

The correlation between the increase in altitude and the decrease in arterial oxygen saturation is well known [21]. In children, blood pressure improves with age; moreover, during early ages, the interindividual variation in arterial oxygen saturation is larger than the variation observed between adult subjects [21]. There is almost no literature about the cardiopulmonary adjustments after acute HA exposure on lowland children and the adolescent population, and most of the available literature refers to highland children and adolescent subjects. 

Physiological responses to maximal and submaximal exercise at HA are well documented, especially with respect to the adult population [22,23]. Nevertheless, almost no studies have been performed on children and adolescent individuals, nor specifically on submaximal exercise under acute HA exposure during naturally (geographical) induced hypoxia. Only a reduced number of publication have studied maximal exercise responses [20]. Submaximal exercise performance is reduced at HA due to lower inspired oxygen pressure for which its main effect is the fall in arterial oxygen content and its availability in the tissues. It is evidenced by a decrease in arterial oxygen saturation measured by pulse oximetry (SpO_2_) [21,24].

Cardiopulmonary exercise test can be safely performed on young children [25]. On the other hand, because the above-mentioned increasing popularization of HA activities with respect to the young population, the understanding of their cardiopulmonary responses during submaximal exercise after acute HA exposure could be vastly valuable. Furthermore, 6 MWT is a validated and reliable test, and it is easy to perform on children [26,27]. 

Limited information is available on cardiorespiratory response at rest and during exercise in children and adolescents at HA. It could be hypothesized that it is correct to transfer the evidence obtained from adults, namely a decrease in performance and greater cardiorespiratory effort. Taking all the previous points into consideration, the main goal of this study was to evaluate basic cardiorespiratory physiological parameters during rest and submaximal exercise responses by means of the six-minute walking test (6 MWT) under natural HA conditions. Secondly, we aimed to identify the signs and symptoms associated with the onset of acute mountain sickness (AMS) and its incidence and evolution after acute HA exposure in children and adolescents.

This study has important implications for the development of AMS prevention strategies and information programs for children and adolescents who will carry out specific leisure activities or training at geographical altitudes. It can also provide relevant information for the prevention and treatment of health complications in child populations assisted in refugee camps located at moderate or high altitudes.

## 2. Materials and Methods

### 2.1. Study Design

This is a retrospective descriptive observational study conducted in Antofagasta at sea level (SL) and Caspana, a small village located at 3300 m above sea level (HA), in the context of a pedagogical Astronomy field trip supported by the University of Antofagasta and the Club de Ciencias Norte. This study was developed during the 2012 academic year.

### 2.2. Participants

Information sessions were held at different schools for parents and students who would participate in an astronomical camp in Caspana. A preliminary survey was applied in order to determine eligibility. Lastly, the children and adolescents who wanted to participate voluntarily in the study and whose parents expressly provided their written informed consent were recruited. Forty-two moderately active and healthy children whom twenty-four were girls and eighteen boys, between 11 and 15 years old, were finally selected among four different schools in Antofagasta, a coastal city (about 400,000 inhabitants) located on Northern Chile. All participants had no history of HA exposure during previous months, and they were born and raised at SL, without surnames of native highlander ancestry. A posteriori subjects were classified according to BMI in three categories: normal weight (NO; eight boys and twelve girls), overweight (OW; seven boys and nine girls) and obese (OB; three boys and three girls).

All participants were free of any chronic disease. Admission criteria included not suffering any heart disease, asthma, epilepsy, acute respiratory disease or musculoskeletal injuries, no history of acute myocardial infarction in the family under 55 years and not having smoking habits. The study strictly adhered to the World Medical Association Declaration of Helsinki with respect to ethical principles for medical research involving human subjects.

### 2.3. Study Procedure

In the first phase of the study, the general characteristics of the selected subjects and any relevant clinical information on symptoms and clinical history were collected during meetings with the parents of the participants.

**Figure 1:**
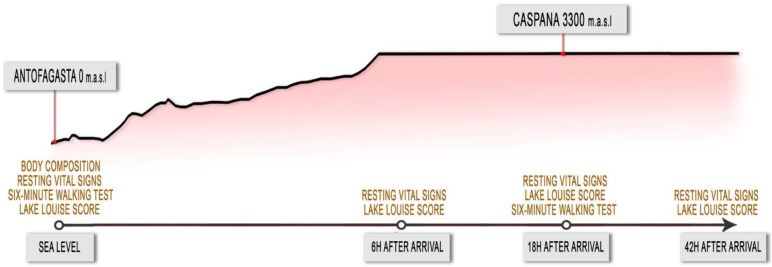
Tests and assessments performed at different altitudes and chronological times.

All 42 participants were submitted to anamnesis, anthropometry and body composition assessment, resting vital signs (RVS) measurements, AMS evaluation and the 6 MWT performance with their subsequent physiological evaluation at SL in Antofagasta just one week before heading up to Caspana, a small village located in the Andean plateau (altitude: 3300 m) about 300 km East from Antofagasta. The students traveled by an urban bus from Antofagasta to Caspana, which took 7 h, making a 3 h stop for lunch and shopping at 2500 m (Calama). Later, 6, 18 and 42 h after arrival to Caspana, all the participants submitted again to RVS measurements and AMS evaluation. A new 6 MWT was performed on the second day, which occurred eighteen hours after the arrival (Figure 1). 

#### 2.3.1. General Characteristics and Body Composition

While in Antofagasta, the gender and age of the subjects were recorded. Body mass was measured using a Seca scale (Seca, Vogel & Halke, GmbH & Co. KG, Hamburg, Germany; precision of 0.1 kg) with participants in underwear and barefoot. Heights were measured with a stadiometer incorporated in the described balance (Seca, Vogel & Halke, GmbH & Co. KG, Hamburg, Germany, precision of 0.1 cm). Waist circumference was quantified by using measuring tape 201® (precision of 0.1 cm), and skinfold (triceps and subscapular) was assessed by using Slim Guide® plycometer (precision of 1 mm).

The percentage of fat mass was estimated by using the Slaughter formula [28]; this equation has the gender of the subject and the summation of triceps and subscapular skin fold in count, measured in millimeters, in order to establish the value of body fat percentage. The Slaughter formula for prepuberal, puberal and postpuberal males and females is commonly used, and previous studies reported that the equation “can be used in male and females to predict body fatness in epidemiological studies or in clinical setting” [29].

Overweight and obesity were objectively identified by using the body mass index (BMI) (kilogram per meter squared) according to the classifications established by the World Health Organization for children between five to nineteen years [30]. We classified the participants on three different BMI groups: normal (NO), lower than twenty-five kg/m^2^, overweight (OW), between 25 and 29.99 kg/m^2^; and obese (OB), higher than 30 kg/m^2^. 

Physical activity status from all subjects was evaluated by using an HAF-INTA questionnaire [31]. The subjects were asked to fill the survey form, which evaluated the following: (1) the total of hours per day lying down; (2) daily hours of minimum activities; (3) number of blocks of houses in the city they walked by daily; (4) daily hours of recreational games; and (5) weekly hours of participation in organized physical activities or sports.

#### 2.3.2. Resting Vital Signs

RVS were assessed while subjects were resting in a sitting position. Heart Rate (HR) and SpO_2_ were measured by using a pulse oximeter (Nonin 8500 M, Nonin Medical Inc, Plymouth, MN, USA) located on subject’s index finger. Systolic and diastolic blood pressure (SBP and DBP) were assessed by using a blood pressure monitor (HEM-742INT, OMRON) placed on the subject’s upper left arm. Finally, the respiratory rate (RR) was directly measured by the investigator, counting the respiratory movements for 60 s.

#### 2.3.3. Six-Minute Walking Test

Two 6 MWTs were performed, the first one was performed at SL and the second one eighteen hours after arrival at 3300 m. Six MWT was performed following the American Thoracic protocol in order to ensure global standardization and also because it is a safe and inexpensive test that requires minimal equipment and time to manage [27]. 

Both tests were performed outdoors on a flat sports field. RVSs were assessed ten minutes before initiating the 6 MWT and immediately after finishing the test. Additionally, RVSs were registered during the recovery phase and three and five minutes after ending the 6 MWT. 

Distance was measured by using a pre-established 30 m circuit, and researchers were in charge of quantifying the number of laps completed by each subject. Dyspnea and fatigue were computed by employing a modified Borg scale (1–10) at the end of each test.

#### 2.3.4. Acute Mountain Sickness Assessment

A Spanish version of the Lake Louise score (LLS) questionnaire was used to evaluate AMS. Occurrences and intensity of headache, gastrointestinal manifestations (nausea, vomiting or poor appetite), fatigue, dizziness and sleep disturbance were recorded using this survey [32]. All these symptoms were scored on scale from zero to three, which indicates absence (0), mild (1), moderate (2) or severe (3) symptoms. 

The LLS questionnaire was assessed at SL and at six, eighteen and forty-two hours after HA exposure. This methodological approach was selected considering the age of the subjects and an easy score system that allowed us to develop an easy communication strategy with the studied subjects. As observed in some previous studies, these points are key aspects for evaluating AMS in children [6]. 

### 2.4. Statistical Analysis

Statistical analysis was performed using SPSS 23.0 software (SPSS Inc., Chicago, IL, USA). Descriptive statistics were calculated for each variable (mean values and standard deviation). The Shapiro–Wilk test was employed to determine if all datasets were normally distributed; based on these results, we further applied parametric or non-parametric tests. 

The children’s general characteristics and body composition were computed by gender. To compare the physiological parameters measured at SL with the same variables obtained at six, eighteen and forty-two hours after HA exposure, ANOVA of repeated samples was performed on those variables following a normal distribution. Variables with a non-normal distribution were compared by using the Freidman test. Furthermore, each variable was compared between genders by using a Student’s *t*-test of related samples, and the Wilcoxon test was used for the comparison of variables with non-normal distributions. Moreover, measures at SL were compared to variables measured six, eighteen and forty-two hours after arriving at Caspana for each BMI group described previously; ANOVA of repeated samples and the Freidman test were used to compare between these groups.

Mean values registered on the two 6 MWTs performed, at SL and at HA, were compared by using the Student’s t-test of related samples and the Wilcoxon test for values with non-normal distributions. We also compared the values from both tests between genders by utilizing the Student’s t-test of related samples. Furthermore, we compared 6 MWT data for the three BMI groups (NO, OW and OB) by applying an ANOVA analysis. 

Finally, AMS was calculated by obtaining the percentage of every variable and their severity. For all statistical tests, an alpha level of 0.05 was selected.

## 3. Results

### 3.1. General Characteristics and Body Composition 

Base line participant’s characteristics are shown on Table 1. There were 18 male participants and 24 females (aged 12.5 ± 1.04 years and 12.5 ± 1.14 years, respectively). The main anthropometric characteristics of the participants are shown in Table 1. 

### 3.2. Resting Vital Signs 

As expected, SpO_2_ values were lower at HA during all range of times compared to the values reported at SL (*p* < 0.001) (Table 2). HR, RR and DBP presented higher values after acute HA exposure compared with SL (*p* < 0.002, *p* = 0.01, *p* = 0.01) (Table 2). No statistical differences on SBP at both altitudes (*p* = 0.366) were detected (Table 2).

When the distinct variables were compared between genders, only RR measured 18 h after HA exposures was statistically significant, where females presented a higher RR than males (*p* = 0.025) (Table 2). 

Additionally, in the comparison of all the values between BMI groups (NO, OW and OB), only HR, SBP and DBP showed statistical differences. In Table 3, we present all resting vital signs segregated by BMI class and gender. After eighteen hours of HA exposure, OB subjects presented higher HR values than NO and OW groups: 113.5 ± 13.71 bpm, 104.95 ± 10.42 bpm and 96.62 ± 8.87 bpm, respectively (*p* = 0.004). 

We also detected statistical differences between the NO group and both the OB and OW groups for SPB and DBP values obtained 42 h after arrival at HA. SBP was much higher in OB participants with 126.16 ± 5.11 mmHg, OW presented mean values of 115.68 ± 14.10 mmHg and NO presented lower values with 112.4 ± 10.67 mmHg (*p* = 0.05) (for more detail, see Table 3). 

DBP was also higher in OB subjects with mean values of 85.16 ± 15.19 mmHg when compared with OW and NO subjects, which presented values of 67.43 ± 7.04 mmHg and 69.25 ± 10.38 mmHg (*p* = 0.02), respectively (see Table 3).

### 3.3. Six-Minute Walking Test

**Figure 2:**
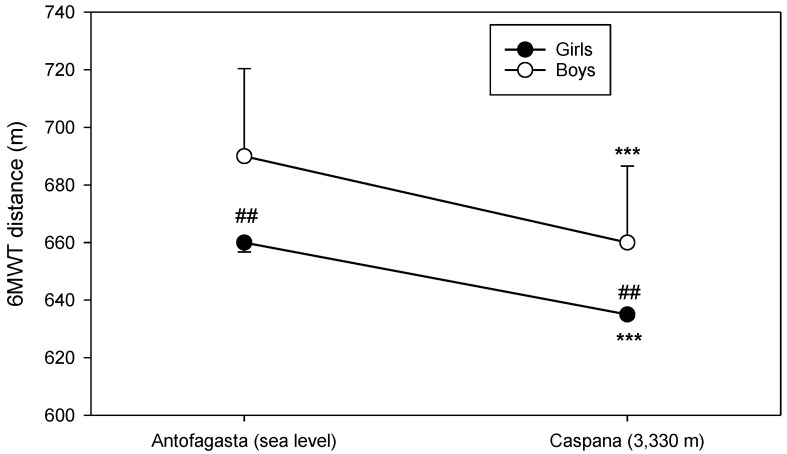
Comparison of 6-min walking distance during SL and HA 6 MWT. Data are expressed as mean ± SD. *** *p* <0.001 HA vs. SL; ## *p* < 0.01 boys vs. girls.

There is a statically significant difference between total distance walked during the 6 MWT at SL (681.18 ± 46.94 m) when compared to HA (654.85 ± 36.78 m) (*p* < 0.001) (Figure 2). As it can also be observed in Figure 2, statistically significant gender differences in 6 MWT distance were evident in both locations: 666.1 m ± 44.0 m at SL vs. 643.6 m ± 34.7 m at HA (*p* < 0.01) for girls and 708.7 m ± 40.2 m at SL vs. 677.3 m ± 30.9 m at HA (*p* < 0.01) for boys. In addition, there was much more heterogeneity in performance in boys than in girls.

**Figure 3:**
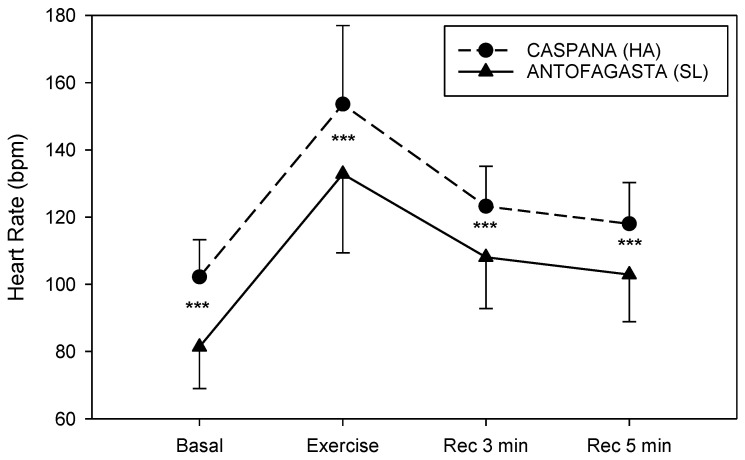
HR responses during 6 MWT at SL (solid line) and HA (dashed line), 10 min before the test (Basal), after test (Exercise) and three (Rec 3 min) and five (Rec 5 min) recovery minutes after the end of the test. Data are expressed as mean ± SD; *** *p* <0.001 vs. SL. SL, sea level; HA, high altitude.

**Figure 4:**
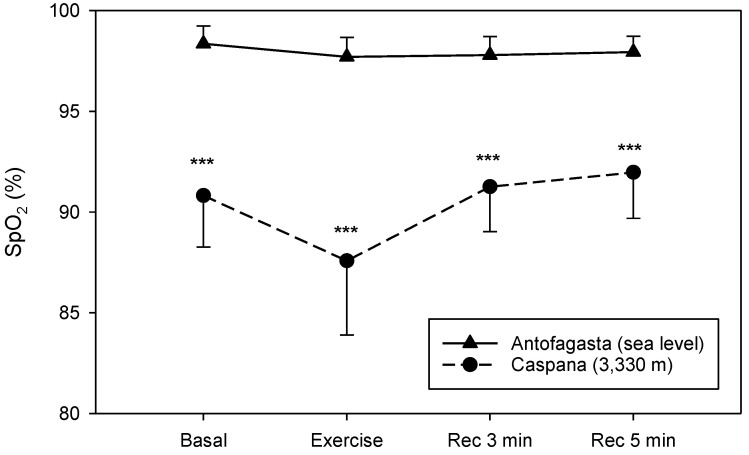
SpO_2_ changes during 6 MWT at SL (solid line) and HA (dashed line) 10 min before the test (Basal), after test (Exercise) and three (Rec 3 min) and five (Rec 5 min) minutes of recovery after the end of the 6 MWT. Data are expressed as mean values ± SD; *** *p* < 0.001 vs SL.

**Figure 5:**
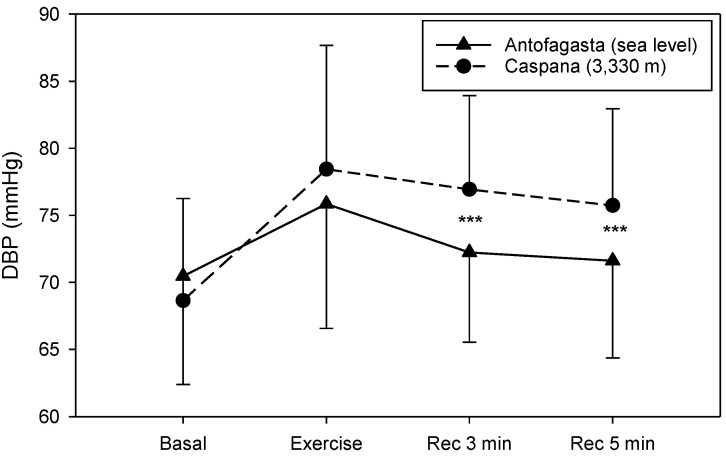
DBP response during 6 MWT at SL (solid line) and HA (dashed line) 10 min before the test (Basal), after test (Exercise) and three (Rec 3 min) and five (Rec 5 min) minutes of recovery after the end of the 6 MWT. Data are expressed as mean values ± SD; *** *p* < 0.001 vs. SL.

**Figure 6:**
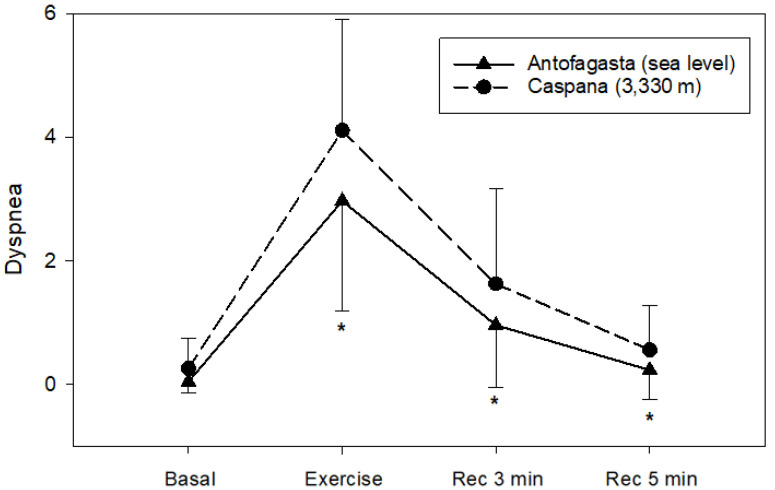
Dyspnea during 6 MWT at SL (solid line) and HA (dashed line) 10 min before the test (Basal), after test (Exercise) and three (Rec 3 min) and five (Rec 5 min) recovery minutes after the test. Data are expressed as mean ± SE * *p* < 0.05 vs. SL.

The contrasts between SL and HA in full view for RVS are presented in Figure 3, Figure 4, Figure 5 and Figure 6. RVSs were measured ten minutes before the 6 MWT (Basal), only a few seconds immediately after the end of the test (Exercise) and after three (Rec 3 min) and five (Rec 5 min) minutes of recovery. Due to the fact that no statistically significant differences between genders were detected for any parameter, data for all subjects were merged. In Figure 3, we present the global average HR at four conditions in the two locations. We observed that, in the four phases of 6 MWT, the HR values were statistically lower at SL when compared to HA (*p* < 0.001).

The values of SpO_2_ at SL do not differ among the four measurements. However, when we compared SpO_2_ levels at SL with the values at HA, we found a statistically significant difference between both altitudes, with a markedly lower SpO_2_ value upon HA exposure (*p* < 0.001) (Figure 4). The observed exercise-induced drops in SpO_2_ are about 3.6%. Moreover, SpO_2_ values presented after 3 min of recovery are similar to baseline levels. We found remarkable low individual variation among young subjects, with standard deviation ranging from 2.5 to 4%.

DBP presented similar values before and immediately after the 6 MWT (*p* = 0.17). On the other hand, 3 and 5 min after the 6 MWT, the values presented at HA were statistically higher than the ones measured at SL (*p* < 0.001) (Figure 5).

Dyspnea response during 6 MWT follows a similar path at both altitudes, but the post-exercise signs are statistically higher at HA in comparison to SL (*p* < 0.05) (Figure 6).

Regarding the comparison between genders of the responses in physiological parameters during the four different states of the 6 MWT performance, we only found statistical dissimilarities on dyspnea values at the end of exercise at HA between boys and girls (*p* = 0.038).

**Figure 7:**
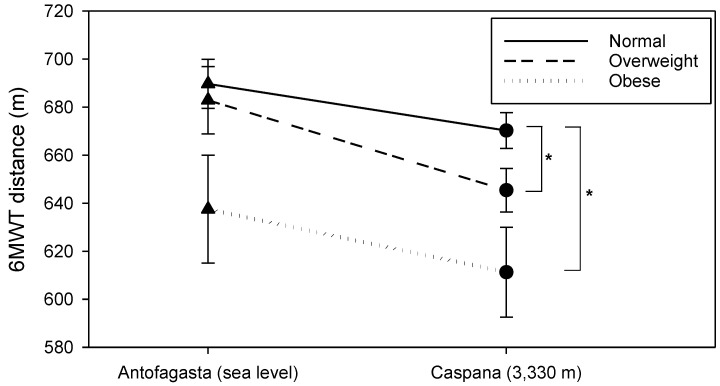
Comparison of the 6-min walking distance during SL and HA 6 MWT on NO, OW and OB participants. Data are expressed as mean ± SE * *p* < 0.05 vs. NO.

However, when comparing the three BMI groups, as we can appreciate in Figure 7, we found a statistical difference between BMI categories in the total distance walked at HA. NO walked 670.27 ± 31.78 m, OW individuals roamed 645.45 ± 30.04 m and OB children reached a distance even lower of 611.25 ± 37.5 m (*p* = 0.05). In addition, we observed lower DBP values in NO participants when compared with OW and OB at the end of the test performed at SL (*p* < 0.001).

### 3.4. Acute Mountain Sickness

At SL, only a minor percentage of participants anecdotally manifested minor symptoms of headache, gastrointestinal problems, fatigue or sleep disturbance. Six hours after HA exposure at 3300 m, the incidence of AMS was 11.9%. In this early phase of altitude acclimatization, a little less than half of the subjects (40.4%) reported mild or moderate headache, and about one in four (26.2%) reported mild or moderate gastrointestinal problems. Just over one-third of the subjects (35.7%) reported mild fatigue, whereas dizziness increased until one out of ten (9.5%). Sleep disturbance was very similar to that reported at SL (Table 4). 

Eighteen hours after arrival, the AMS incidence reached the maximum values, with 19% of AMS affected subjects, although the percentage of incidence of headache, gastrointestinal problems and fatigue decreased when compared with the values presented at 6 h, whereas dizziness remained at similar levels. However, sleep disturbances increased considerably and reached a percentage of almost 62% with mild, moderate, and severe symptoms (Table 4).

Eventually, the values measured forty-two hours after HA exposure showed decreased AMS incidence (9.5%) and lower incidence in terms of the number of affected subjects and severity of symptoms with respect to headache, gastrointestinal problems, fatigue and dizziness. In contrast, sleep disturbance persisted, and its occurrence was barely lower than after eighteen hours, with a prevalence of 54.7% (Table 4).

As the symptoms were generally mild and well tolerated, it was not necessary to apply pharmacological treatment against AMS in any case.

In Table 5, we report the AMS signs for all the subjects and segregated them by sex. We detected only statistically significant differences between both genders for gastrointestinal symptoms after 18 h of HA arrival. For the rest of AMS manifestations and global Lake Louise Score (LLS), no differences were apparent.

Table 6 depicts the AMS signs arranged according to BMI categories. Since no relevant differences between genders were detected (Table 5), we have not considered the intersection between gender and BMI. There is a clear trend for higher grade AMS signs to occur for the higher BMI categories. Headaches were significantly more prevalent in the first hours in OW and OB subjects. On the other hand, differences in dizziness and gastrointestinal manifestations appeared later (after 18 h) and a higher severity with respect to NO subjects persisted after 42 h of HA stay.

## 4. Discussion

Our study provides novel information on the resting and exercise cardiopulmonary responses in children and an adolescent population acutely exposed to geographical hypobaric hypoxia.

### 4.1. Resting Vital Signs

The current study shows that, in children and adolescent populations, the acute exposure to HA notoriously decreased arterial oxygen saturation levels. We also found a low individual variation between young subjects (from eleven to fifteen years), with standard deviation ranging from 2.5 to 4%. A few numbers of studies have been performed by using a comparable design compared to the present study. Our results are consistent with the findings of Screase and colleagues (2009), who measured cardiorespiratory responses and daytime and overnight pulse oximetry in healthy lowland children, reporting mean SpO_2_ values of 88.9% at 3500 m (Namche, Nepal), which are similar to the values we report here (ranging from 89.45% to 90.6%) [33]. Our data are slightly higher than those (from 83 to 89%) described at 3450 m (Switzerland) in children and adolescents [14].

In Chile, Moraga et al. in 2002 reported a correlation between age and altitude with SpO_2_ values. They assessed two different measurements: one at 3500 m and another at 4400 m on adolescents (13–18 years) and children (6–48 months). The values of SpO_2_ in children were 78.8 ± 5.2% at 3500 m, but the values presented by adolescents were clearly the highest (82.2 ± 3.2%). These differences were also reported at 4400 m, where children presented a mean value of 65.3 ± 1.3% and adolescents presented 78.6 ± 2.5%. The authors did not find statistically significant differences in SpO_2_ between adolescents and adults at both altitudes [34]. Subsequently, Moraga and colleagues in 2008 performed a descriptive study at 3500 m on children under 5 years of age. They found SpO_2_ values around 80 ± 2%, lower than the ones we report here (near 90%). The differences between these reports could be explained by age because SpO_2_ values in the youngest children (less than 5 years) after HA exposure could be lower in comparison to our teenagers [35].

A lot of studies have been carried out in children and adolescents who were born and raised at altitude. The individual variation found in our study is consistent with the findings exposed in a recent systematic review related to oxygen saturation in childhood at HA [21]. In addition, in 2007, Weitz and Garruto measured the SpO_2_ values form Tibetan and Han children aged 10 to 14 years at 3200 m. The mean SpO_2_ values they reported were 90%, which are similar to the values we report here (ranging from 89.45% to 90.6%) [36]. 

A previous study reported slight gender differences in SpO_2_ at rest in adults at sea level [37]. However, we could not find gender differences in our teenagers at 3300 m. In contrast, the above-mentioned study performed in Tibet at 3200 m reported that highlander females showed higher SpO_2_ than males [36]. On the other hand, a study performed at 4100 m in Peru on highlander children reported no gender differences in SpO_2_ [38]. Thus, it seems clear that sexual maturation and the well-known effect of estrogen levels on respiratory neuroplasticity and ventilatory control can play a role in the gender differences of SpO_2_ at high altitude. 

We have not found correlations between oxygen saturation and acute mountain sickness, which is in agreement with the studies by Kriemler et al. (2014) [14] and Chen et al. (2012) [39] and contrasting with the studies by Karinen et al. (2010) [40] and Burtscher et al. (2004) [41], who indicate that SpO_2_ is a predictor of AMS. These discrepancies could be explained by the time of exposure, the altitude reached and differences in the ages of the subjects.

Heart rate increased substantially in acute HA exposure, showing the highest values recorded at six hours after the arrival (106.98 ± 14.09 bpm). The next two measures, at 18 and 42 h, showed maintained levels higher than SL (*p* < 0001), in accordance with the findings from Takken and colleagues [20]. Additionally, Moraga et al. showed a mean value of 129 ± 9 bpm, which is higher than the ones we report. It seems that the youngest children (less 5 years) could have an augmented cardiac response to HA exposure when compared with the oldest children and adults [35]. The findings of Moraga et al. in 2002 also confirm higher values of HR at HA compared to SL; additionally, they reported highest HR values at 4400 m than compared to the ones presented at 3500 m (*p* < 0.05) [34].

The values of RR are also higher after the HA exposure to increased ventilation, thus compensating for the lower oxygen availability. Simultaneously, the tachycardia also increases cardiac output, despite the decreased stroke volume caused by acute hypoxia. However, a drop in arterial oxygen saturation is unavoidable due to the shorter transit time of blood in the pulmonary microcirculation (that is due to both the increased cardiac output and the pulmonary hypertension caused by hypoxic vasoconstriction).

Over time, as Naeije concluded in 2010, it seems that in addition to tachycardia and hyperventilation, systemic arterial blood pressure seems to increase temporally in order to compensate for the mentioned lower stroke volume [2]. We found that DBP seems to increase after acute exposure to HA in a similar manner as was also reported by Major et al. in adolescents [42].

Blood pressure increment on the adult population during altitude acclimatization seems to be entirely explained by sympathetic nervous system activation [2]. Consequently, in children and adolescents, the activation of the sympathetic nervous system seems to be similar [43]. Nevertheless, as some authors have manifested, cardiorespiratory response seems to be increased in child populations compared to adolescents after acute HA exposure [34], [35]. The values of SBP did not show a statistical difference between altitudes. In order to compensate the mentioned hypoxemia, the activation of sympathetic efferent pathways seems to be increased.

### 4.2. Six-Minute Walking Test

In our study, the submaximal exercise performance measured through 6-min walking distance was reduced significantly from sea level to high altitude; the main distance walked at SL was significantly longer than the one accomplished at HA (*p* < 0.001), with a drop of 4.3% in boys and 3.5% in girls in the distance walked during 6 MWT at high altitude. The reduction in the distance walked over 6 min is comparable with adult populations [44].

It has been observed that submaximal exercise performance changed during acute exposure to HA [14], and the reduction in submaximal exercise capacity is associated with a decrease in arterial oxygen content as a result of the reduction in the inspired partial pressure of oxygen [2] partly compensated by increases in cardiac output and in local muscle blood flow [14,15]. In fact, in our study, we found an increment on HR at HA compared to SL before and after the test and during the recovery period. An opposite trend was observed in arterial oxygen saturation values; at HA, they were lower than in SL, and this difference was observed in the four phases of the test. The values of DBP during the recovery period were higher at HA. Dyspnea scores were also elevated at HA during all the phases of the test. The perceived difficulty to exercise at a specific intensity will also rise with increasing altitude [22]. Thus, our results confirm that children and adolescents, similar to adults, have lower functional capacities during submaximal exercise after acute HA exposure.

The values of distance walked in the 6 MWT by our participants could be considered in the normal range, and this is similar to that reported in other studies conducted at SL in healthy Chilean children and adolescents [45]. In addition, the gender differences founded in this study are in accordance with previous data in the 6-min walking test performance of children and adolescents at sea level [45,46]. Moreover, in our study, these differences remain at HA, where boys had a higher exercise performance agreeing with previous data in submaximal exercise performance in adults [14] and maximal exercise performance in children [26].

To our knowledge, there have only been a few studies making use of a six-minute walking test to evaluate exercise performance at HA in adults [44], and studies have not been conducted in children and adolescents in this geographical condition. Thus, our results have clinical relevance because it highlights that the six-minute walking test is a safe and well-tolerated procedure and might be considered a useful tool for evaluating cardiorespiratory response to submaximal exercise in children and adolescents at HA. 

On the other hand, we found that the OB and the OW subjects suffered a reduction in their physical capacity to a greater extent than the subjects with normal weight (*p* < 0.05) at HA. Interestingly, the performance differences between NO and OW subjects were not so appreciable at sea level, similar to the findings of Pathare, Haskvitz and Selleck who reported no statistical difference between NO and OW children (*p* = 0.899) at sea level [47]. Moreover, previous studies carried out at sea level using the 6 MWT have shown that overweight and obese children and adolescents had lower physical fitness than NO children [48,49,50]. The differences on submaximal exercise performance at HA in obese children could be explained by the deficient O_2_ uptake at HA and higher demand ventilation, increased work related to breathing, respiratory muscle inefficiency and diminished respiratory compliance given their anthropometric characteristics [51]. 

### 4.3. Acute Mountain Sickness Assessment

AMS incidence after acute HA exposure at 3300 m was between 9.5% and 19% in children and adolescents. Headache and fatigue were the more recurrent symptoms. The range of values observed was lower than the ones reported by Pradhan et al. in 2009, where they reported an incidence of 47.2% in children aged between 3 and 15 years [10]. However, the altitude exposure on this intervention (4380 m) was far higher than the altitude we performed in our study. 

Kriemler and colleagues performed a similar study in 2008, acutely exposing 20 pre-puberal subjects at 3450 m, and they found an AMS incidence of 14% (7 out of 20 participants) [52]. In 2017, Fei-Ying performed an intervention on 11 to 12 years old children at 3100 m, reporting an AMS incidence between 23.4% and 29.4%. Cheng et al. reported a 40.6% of AMS incidence on 197 healthy, non-acclimatized 11 and 12 years old children trekking the round-trip from the trailhead to the summit of Xue Mountain, Taiwan (2179 m to 3886 m), over 3 days [19]. In a similar study, Chan et al. reported a 59% incidence of AMS in a cohort of 96 healthy non-acclimatized children aged 11–12 years who trekked from an elevation of 2600 m to 3952 m in 3 days (Jade Mountain, Taiwan). This incidence was higher than for adults (36%) and was associated with not only altitude but also with recent upper tract respiratory infection (last 7 days) [53]. Dallimore and Rowbotham reported a large range of AMS incidence, between 3.8 and 42.3%, adding that the amount of time the subjects were exposed to HA was large, about six days, allowing probably for full altitude acclimatization. [8]. Studies performed at lower altitudes, reported AMS incidence ranging from 7.3% to 11.3% [54]. These findings suggest that at similar altitudes, the AMS incidence on children population is heterogeneous and can be associated with intrinsic factors such as gender and BMI [35] or extrinsic factors that are probably the same as those identified for adults, such as altitude reached, velocity of ascent, previous upper respiratory tract infection (URTI) and time spent at HA.

In summary, the correlation between the age of the participants and the altitude reached regarding the AMS incidence seems clear. In general, probably due to higher metabolism and oxygen requirements, children seem to present higher AMS incidence than adults and adolescents. Furthermore, as in adults, the incidence of AMS appears to be higher when a higher altitude is reached. 

We must to recognize some methodological limitations: The sample size studied is limited with respect to the potential sample universe, as it depends on the authorization of parents or legal guardians for children to participate in the study. On the other hand, most of them did not provide information about the state of sexual maturation of their children (menarche age or menstruation cycle in girls), and we do not know their real biological age. 

## 5. Conclusions

This study allowed us to quantify the arterial oxygen saturation reduction, even at rest, in adolescents and children from 11 to 15 years old after acute exposure at 3300 m. Other cardiorespiratory parameters such as HR, RR and DBP increased at rest compared to SL. As expected, submaximal exercise performance decreased at HA when compared to SL. Moreover, we reported transitory increases during moderate exercise on HR, DBP and dyspnea during the early phase of high-altitude acclimatization. Six MWT can be a useful and safe tool for evaluating submaximal exercise cardiorespiratory responses in children and adolescents at HA. Interestingly, SpO_2_, RR and HR after arrival to HA, even at rest, but especially during the end of moderate exercise could be considered key parameters for evaluating individual tolerances to acute hypoxia exposure in children and adolescents.

A lower physical performance of the subjects with OW or OB was apparent when compared with NO individuals, which was associated with increased values of HR, SBP and DBP in comparison to their NO mates.

We reported an AMS incidence from 9.5% to 19%, with mild to moderate symptoms.

The evidence found in this study could help health and education professionals and advise parents of children and adolescents traveling to HA locations. Our study demonstrates the importance of early identification of possible symptoms and monitoring of arterial oxygen saturation. An easy-to-perform test (6 MWT) can help to make timely treatment decisions and help, along with preventive measures, in decisions with respect to adjusting ascent rate or increasing scheduled stops during altitude travel in order to minimize adverse symptoms of acute exposure to high altitude in children.

## Figures and Tables

**Table 1:** Mean and SD general characteristics of children by gender.

Anthropometrical Characteristics		*n*	
Age (years)	Boys	18	12.5 ± 1.04
	Girls	24	12.5 ± 1.14
	All	42	12.5 ± 1.09
Body mass (Kg)	Boys	18	56.64 ± 10.53
	Girls	24	53.56 ± 13.76
	All	42	54.93 ± 12.43
Height (m)	Boys	18	1.60 ± 0.11
	Girls	24	1.55 ± 0.07
	All	42	1.57 ± 0.09
Body mass index ^a^ (kg/m^2^)	Boys	18	22.09 ± 2.49
	Girls	24	22.04 ± 4.67
	All	42	22.06 ± 4.16
Waist (cm)	Boys	17	72.9 ± 10.9
	Girls	22	69.8 ± 9.7
	All	39	71.1 ± 10.2
Triceps skin fold (mm)	Boys	17	17.9 ± 7.3
	Girls	22	21.1 ± 6.6
	All	39	19.7 ± 7.0
Subscapular skin fold (mm)	Boys	17	15.9 ± 10.1
	Girls	22	17.2 ± 9.1
	All	39	16.6 ± 9.4
% Fat mass ^b^	Boys	17	25.61 ± 10.39
	Girls	22	29.79 ± 11.24
	All	39	27.97 ± 10.94
Physical activity score (1–7)	Boys	13	4.76 ± 1.69
	Girls	21	4.04 ± 1.32
	All	34	4.32 ± 1.49

^a^ BMI = weight in kilograms divided by square of the height in meters. ^b^ % fat mass = percentage of body fat according to Slaughter formula.

**Table 2:** Resting vital signs in boys and girls at sea level and 6 h, 18 h and 42 h after HA exposure.

Physiological Parameters		*n*	SL	HA		
				6 h	18 h	42 h
spO_2_ (%)	Boys	18	98.0 ± 0.3	89.2 ± 3.9	90.3 ± 2.5	89.8 ± 2.8
	Girls	24	98.1 ± 0.7	88.6 ± 3.9	90.8 ± 3.4	91.2 ± 2.3
	All	42	98.1 ± 0.5	89.5 ± 3.9 ***	90.5 ± 3.0 ***	90.6 ± 2.6 ***
HR (bpm)	Boys	18	82.9 ± 12.4	105.8 ± 15.2	101.8 ± 13.3	99.8 ± 12.8
	Girls	24	83.6 ± 11.2	107.8 ± 13.5	103.9 ± 10.5	102.2 ± 12.8
	All	42	83.3 ± 11.6	107.0 ± 14.1 ***	103.0 ± 11.7 ***	101.2 ± 12.7 ***
RR (bpm)	Boys	18	17.9 ± 3.3	20.3 ± 3.9	20.0 ± 6.8 ^β^	19.3 ± 3.6
	Girls	24	20.1 ± 3.9	24.1 ± 10.0	25.9 ± 9.8	21.9 ± 3.7
	All	42	19.1 ± 3.8	22.5 ± 8.1 *	23.4 ± 9.0 *	20.8 ± 3.8 *
SBP (mmHg)	Boys	18	116.2 ± 12.2	115.5 ± 13.2	116.3 ± 11.2	117.1 ± 14.8
	Girls	24	109.9 ± 10.5	111.2 ± 10.7	112.8 ± 11.1	114.5 ± 10.1
	All	42	112.6 ± 11.5	113.1 ± 11.9	114.3 ± 11.2	115.6 ± 12.3
DBP (mmHg)	Boys	18	63.8 ± 8.2	63.6 ± 9.6	69.9 ± 8.5	72.8 ± 13.1
	Girls	24	61.8 ± 8.2	66.3 ± 8.4	69.1 ± 7.7	69.4 ± 10.2
	All	42	62.7 ± 8.1	66.4 ± 8.8 *	69.4 ± 8.0 *	70.8 ± 11.5 *

Data are expressed as mean values ± standard deviation; ***: *p* < 0.001 vs. SL; *: *p* < 0.05 vs. SL; ^β^: *p* < 0.05 boys vs. girls.

**Table 3:** Resting vital signs stratified by BMI in boys and girls at sea level and 6 h, 18 h and 42 h after HA exposure.

Physiological Parameters			*n*	SL	HA		
					6 h	18 h	42 h
spO_2_ (%)	Boys	NO	8	97.9 ± 0.4	90.0 ± 4.0 ***	90.6 ± 2 ***	90.6 ± 3.4 ***
		OW	7	98.0 ± 0	89.1 ± 3.2 ***	89.7 ± 3.5 ***	89.3 ± 2.1 ***
		OB	3	98.3 ± 0.6	87.3 ± 5.9 ***	90.0 ± 1.2 ***	89.0 ± 2.6 ***
	Girls	NO	12	98.3 ± 0.4	90.3 ± 3.3 ***	90.7 ± 3.2 ***	91.8 ± 2.7 ***
		OW	9	98.0 ± 0.5	89.8 ± 4.9 ***	90.8 ± 3 ***	90.7 ± 2.2 ***
		OB	3	97.3 ± 0.6	86.7 ± 1.5 ***	86.7 ± 4.2 ***	90.0 ± 0.1 ***
HR (bpm)	Boys	NO	8	79.1 ± 12.7	104.8 ± 15.1 ***	102.9 ± 11.5 ***	100.5 ± 12.2 ***
		OW	7	81.7 ± 11.2	100.9 ± 10.4 ***	94.9 ± 9.4 ***	94.4 ± 11.6 ***
		OB	3	96 ± 6.9	120.3 ± 20.5 ***	115 ± 18.3 ***	100.3 ± 14.4 ***
	Girls	NO	12	83.8 ± 11.9	105.7 ± 14.1 ***	106.3 ± 9.9 ***	100.2 ± 15.8 ***
		OW	9	85.9 ± 11.4	109.1 ± 12.2 ***	98 ± 8,7 ***	103.9 ± 10.1 ***
		OB	3	77 ± 7.5 ^β^	112.7 ± 18.7 *** ^β^	112 ± 11.3 ***	105.3 ± 7.0 ***
RR (bpm)	Boys	NO	8	17.3 ± 3.9	20 ± 3.9	18.3 ± 3.5	18.9 ± 3.4
		OW	7	18 ± 3.3	19.3 ± 3.2	18.4 ± 3.2	19.3 ± 4.6
		OB	3	19.3 ± 1.2	23.3 ± 5.9 *	28.3 ± 14 ***	20.7 ± 2.3
	Girls	NO	12	20 ± 4.1	22.3 ± 9.2	23.7 ± 8 ^β^	22.1 ± 2.7 ^β^
		OW	9	19.9 ± 2.3	25.7 ± 11 * ^# β^	26.4 ± 11.1 * ^# β^	20.1 ± 2.9
		OB	3	21 ± 7.8	26.7 ± 12.4 * ^# β^	33 ± 12.1 *** ^# β^	26.3 ± 6.0 * ^# β^
SBP (mmHg)	Boys	NO	8	119.8 ± 14.7	112.8 ± 16.5	114.9 ± 15	113.3 ± 11.6
		OW	7	110 ± 8.5	114.4 ± 9.9	116.1 ± 8.2	115.9 ± 18.9
		OB	3	121 ± 7.9	125.3 ± 7.1	120.3 ± 7.2	130 ± 2.0 *
	Girls	NO	12	105.8 ± 10.7	106.7 ± 9.3	112.6 ± 12.4 *	111.8 ± 10.5 *
		OW	9	112.4 ± 9.8	115.7 ± 10.5	113.3 ± 11	115.6 ± 10.3
		OB	3	118.7 ± 2.5	117.3 ± 10.7	111.7 ± 8.3	122.3 ± 4.2 *
DBP (mmHg)	Boys	NO	8	63.4 ± 8.6	65 ± 7.9	72 ± 11.3 *	70.5 ± 7.5 *
		OW	7	61.1 ± 7.5	66.3 ± 12.1	66.3 ± 5.4	67.4 ± 7.5 *
		OB	3	71.3 ± 5.9	71.7 ± 8.7	72.7 ± 2.1	91.3 ± 21.5 *
	Girls	NO	8	59.3 ± 6.4	64.2 ± 8.8	66.7 ± 9	68.4 ± 12.2 *
		OW	7	64.2 ± 10.5	68.6 ± 7.7	70.8 ± 6.0	67.4 ± 7.1
		OB	3	64.7 ± 4.5 ^β^	67.7 ± 9.5	73.7 ± 4.0 *	79 ± 1.0 *** ^#^
LLS	Boys	NO	8	-	0.8 ± 0.9	0.9 ± 1	1.4 ± 1.6
		OW	7	-	1.0 ± 0.8	1.0 ± 1.3	0.4 ± 0.5
		OB	3	-	1.7 ± 1.5	2.3 ± 1.2	1.3 ± 1.2
	Girls	NO	12	-	1 ± 1.2	0.9 ± 0.8	0.9 ± 0.8
		OW	9	-	1.6 ± 1.6	1.6 ± 1.8	2.1 ± 1.8
		OB	3	-	2.7 ± 1.2	2 ± 1.7	1.3 ± 0.6

Data are expressed as mean values ± standard deviation; ***: *p* < 0.001 vs. SL; *: *p* < 0.05 vs. SL; ^#^: *p* < 0.05 among BMI groups; ^β^: *p* < 0.05 boys vs. girls.

**Table 4:** Acute Mountain sickness assessment according to Lake Louise score (LLS).

	Antofagasta (SL)				Caspana (HA 3300 m)											
					6 h				18 h				42 h			
Lake Louise Score	S1	S2	S3	%	S1	S2	S3	%	S1	S2	S3	%	S1	S2	S3	%
Headache	1	0	0	2.4	15	2	0	40.4	10	1	0	26.2	4	1	0	11.9
Gastrointestinal	1	0	0	2.4	9	2	0	26.2	8	1	0	21.4	3	1	0	9.5
Fatigue	4	0	0	9.5	14	1	0	35.7	7	2	0	21.5	6	1	0	16.7
Dizziness	0	0	0	0	4	0	0	9.5	3	1	0	9.5	2	0	0	4.8
Sleep disturbances	3	0	0	7.1	3	0	0	9.5	16	11	1	61.9	14	9	0	54.7
AMS incidence	N = 0			0	N = 5			11.9	N = 8			19	N = 4			9.5

SL, sea level; HA, high altitude. The Lake Louise score (LLS) is expressed as the number of subjects who declared symptoms as the following: S1: mild symptoms; S2: moderate symptoms; and S3: severe symptoms. AMS occurrence was considered in subjects with LLS ≥ 3 associated with headache. Data in bold characters represent the percentage with respect to the total sample studied (*n* = 42).

**Table 5:** AMS signs according to gender at the different times.

Lake Louise Score		*n*	SL	HA		
				6 h	18 h	42 h
Headache	Boys	18	0.00 ± 0.0	0.22 ± 0.4	0.11 ± 0.3	0.0 ± 0.0
	Girls	24	0.40 ± 0.2	0.54 ± 0.7	0.25 ± 0.5	0.17 ± 0.5
	All	42	0.20 ± 0.2	0.58 ± 0.1	0.19 ± 0.5	0.10 ± 0.4
Gastrointestinal	Boys	18	0.00 ± 0.0	0.17 ± 0.4	0.06 ± 0.2	0.13 ± 0.5
	Girls	24	0.40 ± 0.2	0.38 ± 0.6	0.38 ± 0.6 *	0.11 ± 0.3
	All	42	0.21 ± 0.2	0.29 ± 0.5	0.24 ± 0.5	0.12 ± 0.4
Fatigue	Boys	18	0.17 ± 0.4	0.39 ± 0.5	0.44 ± 0.7	0.11 ± 0.3
	Girls	24	0.39 ± 0.2	0.33 ± 0.6	0.13 ± 0.3	0.29 ± 0.6
	All	42	0.10 ± 0.3	0.36 ± 0.6	0.26 ± 0.5	0.21 ± 0.5
Dizziness	Boys	18	0.00 ± 0.0	0.17 ± 0.4	0.22 ± 0.6	0.06 ± 0.2
	Girls	24	0.00 ± 0.0	0.40 ± 0.2	0.04 ± 0.2	0.08 ± 0.3
	All	42	0.00 ± 0.0	0.10 ± 0.3	0.12 ± 0.4	0.07 ± 0.3
Sleep disturbances	Boys	18	0.11 ± 0.3	0.17 ± 0.4	0.72 ± 0.82	0.72 ± 0.8
	Girls	24	0.00 ± 0.0	0.40 ± 0.2	1.04 ± 0.9	0.75 ± 0.8
	All	42	0.05 ± 0.2	0.10 ± 0.3	0.90 ± 0.9	0.74 ± 0.8
AMS (LLS)	Boys	18	2.01 ± 0.0	1.94 ± 0.2	1.94 ± 0.2	1.94 ± 0.2
	Girls	24	1.80 ± 0.6	1.96 ± 0.2	1.88 ± 0.3	1.92 ± 0.3
	All	42	1.90 ± 0.4	1.95 ± 0.2	1.90 ± 0.3	1.93 ± 0.3

Data are expressed as mean values ± standard deviation; *: *p* < 0.05 between boys and girls.

**Table 6:** AMS manifestations according to BMI categories at the different times.

BMI		*n*	SL	HA		
				6 h	18 h	42 h
Headache	N	20	0.00 ± 0.0 *	0.25 ± 0.4 *	0.10 ± 0.3	0.05 ± 0.2
	OW	16	0.00 ± 0.0 *	0.38 ± 0.6 *	0.19 ± 0.5	0.29 ± 0.5
	O	6	0.17 ± 0.4 *	1.00 ± 0.6 *	0.50 ± 0.6	0.04 ± 0.3 *
Gastrointestinal	N	20	0.05 ± 0.2	0.30 ± 0.6	0.15 ± 0.4 *	0.10 ± 0.0 *
	OW	16	0.00 ± 0.0	0.31 ± 0.6	0.31 ± 0.6 *	0.01 ± 0.8 *
	O	6	0.00 ± 0.0	0.17 ± 0.4	0.33 ± 0.5	0.50 ± 0.0
Fatigue	N	20	0.10 ± 0.3	0.30 ± 0.6	0.10 ± 0.3	0.15 ± 0.5
	OW	16	0.06 ± 0.3	0.44 ± 0.6	0.31 ± 0.6	0.31 ± 0.6
	O	6	0.17 ± 0.4	0.33 ± 0.5	0.67 ± 0.8	0.17 ± 0.4
Dizziness	N	20	0.00 ± 0.0	0.17 ± 0.2	0.10 ± 0.3 *	0.00 ± 0.0
	OW	16	0.00 ± 0.0	0.50 ± 0.3	0.00 ± 0.0 *	0.06 ± 0.3 *
	O	6	0.00 ± 0.0	0.13 ± 0.4	0.51 ± 0.8 *	0.33 ± 0.5 *
Sleep disturbances	N	20	0.10 ± 0.3	0.15 ± 0.4	0.91 ± 0.9	0.65 ± 0.8 *
	OW	16	0.00 ± 0.0	0.06 ± 0.3	0.88 ± 1.1	0.69 ± 0.8
	O	6	0.00 ± 0.0	0.00 ± 0.0	1.00 ± 0.0	1.17 ± 0.1
AMS	N	20	1.90 ± 0.5	2.00 ± 0.0	1.95 ± 0.2	2.00 ± 0.3
	OW	16	1.88 ± 0.5	1.94 ± 0.3	1.94 ± 0.3	1.88 ± 0.4
	O	6	2.01 ± 0.1	1.83 ± 0.4	1.67 ± 0.5	1.83 ± 0.3

Data are expressed as mean values ± standard deviation; *: *p* < 0.05 between BMI groups.

## Data Availability

The raw data supporting the conclusions of this article will be made available by the corresponding author, without undue reservation.
